# Patterned Biolayers of Protein Antigens for Label-Free Biosensing in Cow Milk Allergy

**DOI:** 10.3390/bios13020214

**Published:** 2023-02-01

**Authors:** Augusto Juste-Dolz, Estrella Fernández, Rosa Puchades, Miquel Avella-Oliver, Ángel Maquieira

**Affiliations:** 1 Instituto Interuniversitario de Investigación de Reconocimiento Molecular y Desarrollo Tecnológico (IDM), Universitat Politècnica de València, Universitat de València, 46022 Valencia, Spain; 2 Departamento de Química, Universitat Politècnica de València, 46022 Valencia, Spain

**Keywords:** diffraction, grating, microcontact printing, casein, bovine serum albumin, β-lactoglobulin, covalent, immunoglobulin G, dairy, beef

## Abstract

This paper focuses on creating one-dimensional diffractive grooved structures of antigen proteins on glass substrates for the label-free detection of antibodies to dairy allergens. In particular, the fabrication of protein structures is carried out by combining microcontact printing with physisorption, imines coupling, and thiol-ene click chemistry. The work first sets up these patterning methods and discusses and compares the main aspects involved in them (structure, biolayer thickness, functionality, stability). Homogeneous periodic submicron structures of proteins are created and characterized by diffractive measurements, AFM, FESEM, and fluorescence scanning. Then, this patterning method is applied to proteins involved in cow milk allergy, and the resulting structures are implemented as optical transducers to sense specific immunoglobulins G. In particular, gratings of bovine serum albumin, casein, and β-lactoglobulin are created and assessed, reaching limits of detection in the range of 30–45 ng·mL^−1^ of unlabeled antibodies by diffractive biosensing.

## 1. Introduction

Developing new strategies for patterning biological layers entails nowadays a major scientific interest that leads to appealing bioanalytical developments in a wide range of scenarios [1,2,3,4]. Microcontact printing (µCP), often known also as soft lithography, has emerged as a practical method to create functional patterns of biomolecules [5,6]. This is a widely used technique thanks to its simplicity, versatility, and minimal requirements for microfabrication facilities.

**Figure 1:**
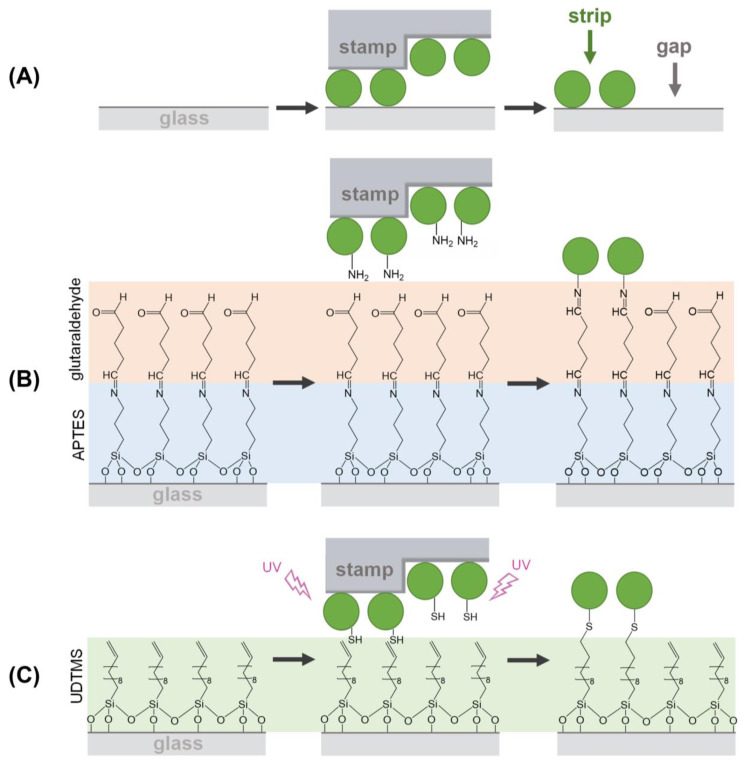
Schemes of the µCP routes investigated in this study for patterning proteins by combining µCP with: (**A**) physisorption, (**B**) imine coupling reaction, and (**C**) thiol-ene click reaction.

As schematized in Figure 1A, µCP relies on a selective transfer of biomolecules using patterned stamps made of an elastomer (typically PDMS), which is usually obtained by replica molding from a pre-patterned master [7,8]. The biomacromolecules incubated on the stamp during the inking stage become adsorbed on the PDMS surface, and in the subsequent stamping step, they are only transferred in the contact areas, thus obtaining a pattern on a solid substrate. The patterning of biological species by µCP is typically mediated by physisorption (Figure 1A) [9,10]. In this case, the transfer efficiency of the biomolecules ultimately depends on their affinity for the substrate material, which should be higher than that for the stamp [11].

µCP can be customized by incorporating functional groups tailored to undergo linking reactions between the inked biomolecules and the surface of the substrates. This strategy introduces patterning alternatives where the transfer during the stamping stage is mainly driven by chemical reactions [12,13,14,15]. Developing ways to pattern and control even smaller structures is a crucial aspect of the worldwide focus on nanoscience and nanotechnology. However, despite the extensive attention that µCP has received in the scientific literature, to the best of our knowledge, the implementation of covalent chemistries for patterning submicron (from 0.1 to 1 µm) structures of biomacromolecules remains unexplored [16,17,18,19,20,21].

Within the biosensing scenario, the fabrication of protein nanostructures points towards exploiting new light-matter phenomena to transduce biorecognition events [22,23,24,25]. The increasing incidence of chronic and inflammatory diseases such as allergies supports the development of this kind of nanobiosensors [26]. Particularly, allergies to dairy products are acquiring a special concern since they are prevalent food products in human nutrition, representing 14% of the caloric intake in developed countries [27]. Among all the constituents present in dairy products, casein and β-lactoglobulin (BLG) are important proteins in cow milk allergy [28,29]. Bovine serum albumin (BSA) is a relevant protein in beef allergy that is also involved in the allergic response to cow milk [29,30]. Along these lines, in addition to their general relevance in immunosensing, immunoglobulins G (IgGs) may also play a key role in allergic diseases [30,31,32,33]. IgGs are considered to be part of the normal immune response to foreign antigens [34]. Although the evaluation of specific IgGs in serum has not yet been revealed as having a predictive value in food allergy diagnosis [34], the relation between the IgGs and IgEs levels can be employed to distinguish between persistent and transient food allergies, and it is also considered a predictor for future tolerance [35]. Moreover, the higher levels of IgGs in IgE-mediated allergic processes, together with their long persistence in serum, make them an interesting alternative to study allergies to cow milk. 

This work firstly focuses on key aspects in the fabrication of submicron diffractive patterns of protein allergens by µCP on glass surfaces. The role of UV-ozone treatments typically employed to improve protein transfer and the implementation of different patterning chemistries are thoroughly explored, compared, and characterized. Then, from these results, diffractive gratings of three important proteins in cow milk allergy (BSA, casein, and BLG) are fabricated and employed as optical transducers for biosensing. Along these lines, the biorecognition events between the patterned antigens and their target IgGs in solution are characterized and sensed in a label-free format. Insights into the selectivity of the resulting biosensing system, and its potential to avoid non-specific binding issues in the analysis of serum samples, are also provided in this study.

## 2. Materials and Methods

### 2.1. Materials

Bovine serum albumin (BSA), whole antiserum with anti BSA antibodies produced in rabbit (antiBSA IgG, 3.8 mg·mL^−1^ of specific IgGs), casein and BLG from bovine milk, human serum (male, AB plasma), polysorbate 20 (Tween 20), (3-aminopropyl)triethoxysilane (APTES), glutaraldehyde, N-hydroxysuccinimide (NHS), N-(3-Dimethylaminopropyl)-N′-ethylcarbodiimide (EDC), and ethanolamine were supplied by Sigma-Aldrich (Madrid, Spain). Anticasein antibodies (0.33 mg·mL^−1^ of specific IgGs) and antiBLG antibodies (1 mg·mL^−1^ of specific IgGs) were from Ingenasa (Madrid, Spain). 10-undecenyltrimethoxysilane (UDTMS) was purchased from Gelest (Morrisville, Pennsylvania, USA). Toluene was from Scharlau (Barcelona, Spain). Polydimethylsiloxane (PDMS) Sylgard 184 was acquired from Dow Corning (Wiesbaden, Germany) and glass slides (standard line, 25 × 75 × 1.2 mm) were from Labbox (Mataró, Spain). Alexa Fluor 647 antibody labeling kit was from Thermo Fischer (Waltham, MA, USA). The silicon grooved structure (555.5 nm period, 100 nm groove depth, duty cycle 50%) used as a master for µCP was supplied by LightSmyth (Eugene, OR, USA). Sodium phosphate buffer (PBS, 8 mM Na_2_HPO_4_, 2 mM KH_2_PO_4_, 137 mM NaCl, 2.7 mM KCl, pH 7.4) and PBS-T (PBS with polysorbate 20 0.05% *v/v*), were prepared with ultrapure water (Milli-Q, Millipore Iberica, Darmstadt, Germany) and filtered with 0.2 μm polyethersulfone membranes (Merck, Darmstadt, Germany).

### 2.2. PDMS Stamps

To create the PDMS stamps, the Sylgard 184 elastomer was mixed with its curing agent (10 to 1 mix ratio). This mixture was thoroughly homogenized, dispensed onto the structured side of the silicon master, degassed in a vacuum chamber for 30 min, and then polymerized overnight at 60 °C. Next, the cured PDMS was peeled off from the master and cut into 5 × 5 mm squared pieces. The resulting stamps were sonicated three times for 5 min in ethanol (30% in MilliQ water) and dried under an air stream before use.

For the ozone treatment, PDMS stamps were oxidized in an ozone atmosphere generated with a 347 nm UV lamp (UVOH 150 lab, FHR Anlagenbaum GmbH, Ottendorf-Okrilla, Germany).

### 2.3. Characterization

Static contact angles were measured to evaluate the surface wettability of the glass and PDMS surfaces under study. For that, an optical tensiometer (Attention Theta Lite, Biolin Scientific, Sweden) was employed to calculate the values of purified water droplets (4 µL). Averaged and standard deviation values were calculated from three replicates measured for 10 s.

Fluorescence measurements were carried out by incubating target IgGs labeled with a fluorophore (Alexa Fluor 647) onto the protein patterns. A custom setup consisting of a charge-coupled device camera (Retiga, EXi, Qimaging Inc., Burnaby, BC, Canada) as the detector, and light-emitting diodes (Toshiba TLOH157P, Tokyo, Japan) as the light source was employed to acquire the fluorescence images. The image analysis for fluorescence quantification was performed with the GenePix Pro 4.0 software (Molecular Devices, San José, CA, USA). Averaged and standard deviation values were calculated from the three parallel measurements of each condition.

The topographic characterizations were performed by Field Emission Scanning Electron Microscopy (FESEM) and atomic force microscopy (AFM). For FESEM measurements, PDMS stamps were first coated with a 10 nm layer of palladium using a high vacuum coater (Leica EM MED020, Leica Microsystems, Wetzlar, Germany) and then they were analyzed using a ZEISS ULTRA-55 scanning electron microscope (ZEISS, Oxford instruments). AFM measurements of both PDMS stamps and protein patterns were performed with a Bruker Multimode 8 microscope (Bruker, Billerica, MA, USA) using RFESPA probes (MPP-21120–10, Bruker). Averaged cross-section profiles were calculated from the resulting AFM images employing the Nanoscope Software. A second-order polynomial flattening was applied to each image and the height was averaged along the longitudinal direction of the pattern strips. The period of the structures was calculated as the sum of the average width of the strips and the average width of the gaps between them. The duty cycle was calculated as the average width of the strips, multiplied by 100, and divided by the period.

### 2.4. Diffractive Measurements

In this study, diffractive measurements were performed to assess the structural features of the PDMS stamps and the protein patterns. This detection principle (herein called diffractive biosensing) requires that the measured structures are periodic and that they fulfill the Bragg condition to diffract an incident laser beam. In this case, one-dimensional diffraction grating structures with a submicron-range periodicity (555 nm) were employed, since they split incident laser beams of visible light into multiple beams (called diffraction orders) distributed in a single row, which simplifies the detection setup and the optical measurements. The intensity of the diffraction orders decreases if the periodic features of the measured structure worsen. Moreover, this intensity increases together with the contrast in height and/or refractive index between the strips and gaps of the grooved structures (Figure 1A). Along these lines, the binding events between the patterned protein and specific antibodies increase the amount of biological matter in the grating strips, which enhances this contrast and increments the intensity of the diffraction orders. As a result, this detection principle provides useful information on the thickness and periodic features of the measured structures and enables quantifying biorecognition assays.

**Figure 2:**
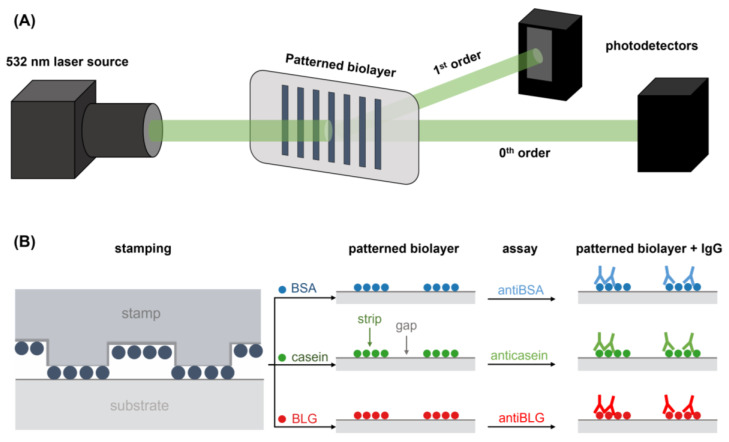
Schemes of (**A**) the optical setup employed to perform the diffraction measurements and (**B**) the fabrication by µCP of the BSA, casein, and BLG patterns employed for the quantification of specific IgG.

The diffractive response was evaluated using a custom optomechanical setup arranged as illustrated in Figure 2A. It comprises a collimated and attenuated (95%) 532 nm diode laser (100 mW, MGL-III-532, CNI, Changchun, China), and a holder which clamps the diffractive samples (PDMS stamps and protein patterns on glass slides) and fixthem to be orthogonally irradiated by the laser beam. The setup also includes a monochromatic CMOS camera (1 ms of exposure time, Edmund eo-1312m, York, UK) and planar silicon photodiodes (SLC-61N2, Silonex Inc., Montreal, QC, Canada) to measure the intensity of the zeroth and the first diffraction orders coming from the diffractive structures. The diffraction efficiency was calculated as the light intensity of the first diffracted order divided by the light intensity of the incident laser beam. Averaged and standard deviation values were calculated from the measurement of three different replicates of each sample.

### 2.5. Surface Functionalization

The glass slides used as substrates were washed by sonication (5 min) in ethanol (30% in milli-Q water) and dried under a stream of air. To functionalize their surface, they were irradiated with a 347 nm UV-lamp (UVOH 150 lab, FHR Anlagenbaum GmbH, Ottendorf-Okrilla, Germany) for ten minutes to generate hydroxyl groups (Figure S1A). Then, the hydroxyl-activated substrates were immersed into 1% (*v/v*) solutions of organosilanes (UDTMS or APTES) in toluene for 30 min and under orbital agitation. After silanization, the substrates were rinsed with acetone and air-dried. Thereafter, substrates were cured at 80 °C for 30 min, rinsed with acetone, and dried as before.

Before protein patterning, aminated substrates (functionalized with APTES) were immersed into a 2.5% solution of glutaraldehyde in PBS for 30 min (Figure S2) [16]. After immersion, the substrates were rinsed with MilliQ water and air-dried.

### 2.6. Protein Patterning

Submicron structures of BSA, casein, and BLG were fabricated by microcontact printing (Figure 2B). The inking and stamping conditions were adapted from previous studies [9]. For the inking, solutions of BSA, casein, and BLG in PBS (250 µg·mL^−1^, 40 µL) were incubated for 160 min at room temperature (22 °C) onto the structured side of the stamps. Then, the stamps were rinsed with Milli-Q water and dried under a stream of air. To perform the stamping stage, the structured side of the inked stamps was placed in contact with the surface of the glass substrates (unmodified or functionalized) for 20 min. In the substrates modified with UDTMS, the stamps were then irradiated with the UV lamp during the stamping stage to conduct the thiol-ene click reaction. Finally, the stamps were removed, and the substrates were rinsed with MilliQ water and dried as before.

### 2.7. Biorecognition Assays

Custom adhesive polymeric masks were adhered to the glass substrates to create open cells to incubate 50 µL of IgG solutions in PBS-T to perform the assays (Figure 2B). In addition, Alexa 647-labeled anti-BSA rabbit IgGs were incubated to assess the protein patterns by fluorescence. After 20 min of incubation, the substrates were rinsed with PBS-T and MiliQ water and dried under a stream of air. Limits of detection (LOD) and quantification (LOQ) were calculated from the trend fitted to the experimental data of the dose-response curves. The LOD was determined as the concentration associated to the mean signal of ten blank measurements plus three times their standard deviation. The LOQ was determined as the concentration related to the mean signal of ten blank measurements plus ten times their standard deviation. The linear range was calculated as the concentration interval above the LOQ that displays a correlation coefficient value (R^2^) of at least 0.99 when the experimental results are fitted to a linear trend.

## 3. Results and Discussion

### 3.1. PDMS Activation

The hydrophilicity of the PDMS surface is an important aspect of the performance of µCP [21], and the UV-ozone treatment of PDMS is a well-established strategy to modulate this parameter. It is reported that the ozone produced by UV-irradiation of molecular oxygen reacts with the non-polar methyl groups at the PDMS surface and increases its hydrophilicity by introducing polar SiO_x_ groups [36,37]. The incorporation of this oxidation stage has demonstrated to be an effective way to improve the transfer rate of biomacromolecules for creating biolayer patterns constituted by features above the micron range [38]. However, this UV irradiation involves critical aspects to pattern biomacromolecules by µCP in the submicron range and below. In addition, characterizing this surface transformation is important to introduce UV-mediated chemical couplings in µCP, as investigated in the next section.

**Figure 3:**
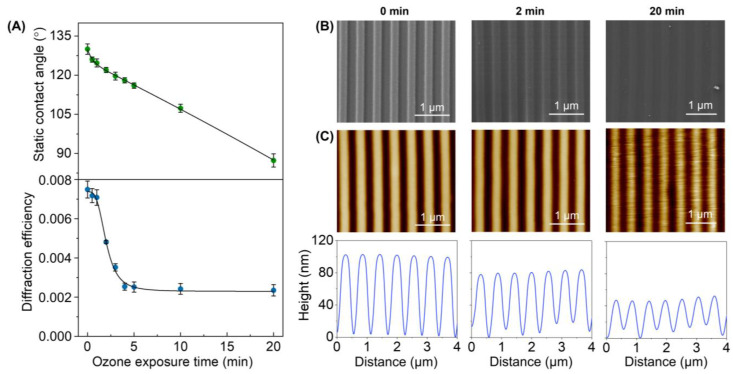
Effect of the UV-ozone exposure on the PDMS grooved structure. (**A**) Evolution of the surface hydrophobicity (top) and the diffractive response (bottom) for increasing exposure times. (**B**) FESEM images (see Figure S3 for larger scans). (**C**) AFM images (top) and their corresponding height profiles (bottom).

PDMS stamps were created from a grooved silicon master defined by a period of 555.5 nm, a groove depth of 100 nm, and a duty cycle of 50%. To assess the effect of the UV-ozone treatment on these submicrometric patterns, the PDMS stamps were exposed to different irradiation times, and the resulting surfaces were characterized by different techniques. First, we studied the static contact angle of water droplets on the grooved surface to quantify the changes in surface hydrophilicity. As shown in Figure 3A (top), the contact angle of the PDMS stamps decreases linearly from 130 ± 2° to 87 ± 3° when the exposure time increases. This trend indicates that the hydrophilicity of this surface increases together with its exposure to ozone, as is expected to improve the protein transfer by µCP. Note that the grooved structure of this surface confers a higher contact angle (130 ± 2°) than that reported in the literature for untreated PDMS (105°) [36].

Then, the effect of this treatment on the submicron features of the PDMS was assessed by diffractive measurements. The grooved pattern on these PDMS surfaces diffracts when irradiated with a 532 nm laser beam, and the efficiency of this diffraction ultimately depends on the features that define the grooved pattern (period, depth, homogeneity, etc.). Therefore, changes in the overall structural features can be monitored through the diffraction efficiency. The experimental results (Figure 3A, bottom) show that the stamps keep their original surface topography for exposure times of up to one minute since the PDMS structures display the maximal diffractive response. However, a dropping trend in the diffraction efficiency is observed beyond this exposure time, which indicates a substantial modification of the pattern.

The FESEM images of the resulting PDMS stamps reveal that they keep their structural homogeneity even after 20 min of exposure (Figure 3B), and both the period and the duty cycle correlate well with the original values (Table S1). However, these images suggest a decrease in the groove depth, which was confirmed and quantified by AFM. As observed in Figure 3C, the grooves on untreated PDMS stamps display a depth of 99 ± 1 nm. However, the UV-ozone treatment progressively decreases this depth, reaching a value of 39 ± 6 nm at 20 min (Table S1).

**Figure 4:**
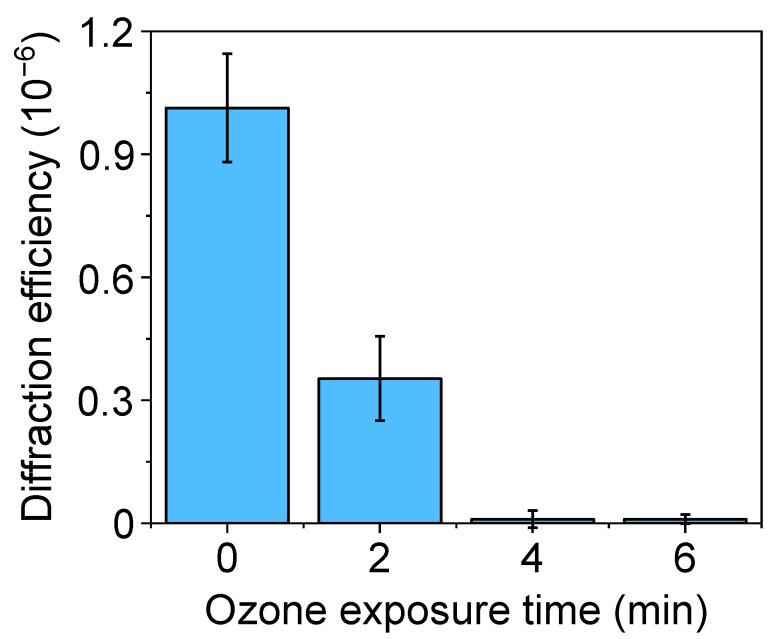
Diffraction efficiency of protein patterns fabricated by µCP on glass, with PDMS stamps treated by different UV-ozone exposure times before the inking.

Therefore, these results reveal that the UV-ozone exposure times that introduce substantial hydrophilicity changes that favor the protein transfer, also lead to structural losses on the submicron relief needed for the biolayer patterning. This depth decrease favors undesired roof collapse deformations during the stamping stage [39], and its negative effect on the resulting submicron patterns of biolayers is proved in the next section (Figure 4). From these results, exposure times below two minutes were selected in the next steps of this study.

### 3.2. Patterning Chemistries

In addition to classical µCP strategies based on physisorption, alternative chemical couplings can also be implemented to attach the patterned biomolecules to the host substrate. In this section, we explore and compare different physisorption and covalent ways to create submicron one-dimensional patterns of proteins by µCP, using BSA as a representative model system.

**Figure 5:**
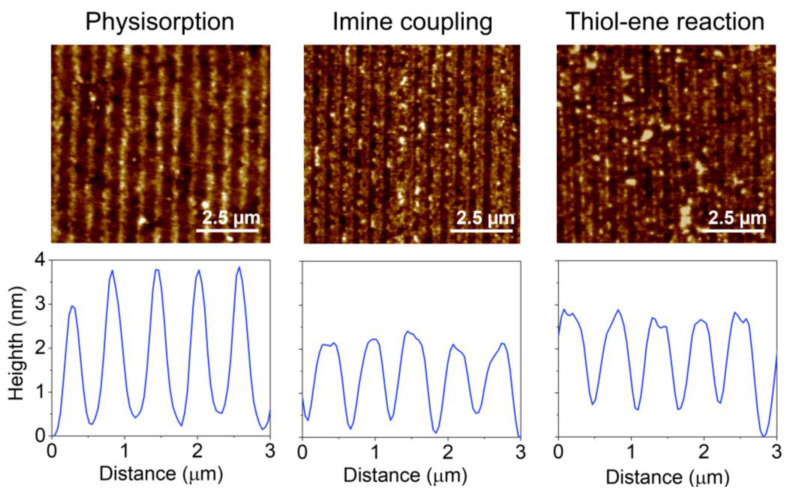
AFM images and height profiles of protein patterns fabricated by µCP combined with physisorption, imine coupling, and thiol-ene reaction.

In physisorption, the transfer of inked proteins is mainly driven by weak forces such as electrostatic and Van der Waals interactions [18]. As reported above, UV-ozone treatments of the PDMS may lead to structural changes that compromise the µCP performance in submicron patterning. In addition to decreasing the depth of the grooves (Figure 3C), the submicron BSA patterns obtained with treated stamps deteriorate when the UV-ozone exposure time increases, since their diffraction efficiency decays drastically with the exposure time (Figure 4). From these results, we addressed this patterning using untreated stamps. As shown in Figure 5, a grooved structure that matches the structural features of the employed stamp is obtained (Tables S1 and S2). The resulting thickness of the patterned proteins (3.3 ± 0.4 nm) suggests a surface density close to a monolayer in the grating strips and agrees with the magnitude of the diffracted efficiencies measured (Figure 4) [40,41].

Then, we explored the combination of µCP with the imine formation between amines and aldehydes to covalently attach submicron patterns of proteins on the surfaces (Figure 1B). First, we observed that the inking stage with organosilanes degrades the submicron structure of the stamp (Figure S4). Therefore, BSA proteins were stamped on glass substrates previously treated with APTES, together with glutaraldehyde as a crosslinking reagent (Figure 1B). As shown in Figure 5, the aimed striped pattern is obtained by this approach, whose averaged strip thickness (2.0 ± 0.3 nm) indicates a slightly lower surface density of the patterned proteins than that obtained by physisorption (Table S2). A potential biosensing drawback of this imine coupling is that part of the aldehyde groups remains active after the patterning, and this issue is successfully solved by treating the protein patterns with aminated blocking agents before the incubation of the sample (Figure S5).

**Figure 6:**
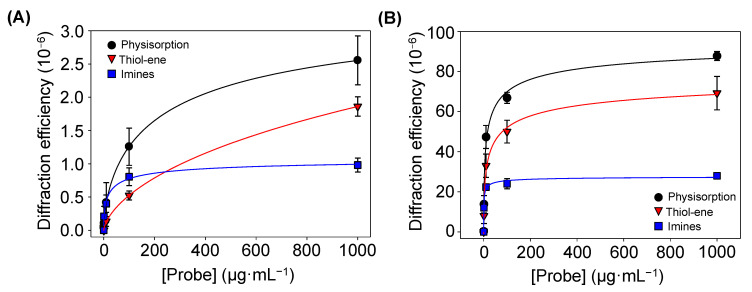
Diffraction efficiencies of the BSA patterns (**A**) before and (**B**) after incubating specific antiBSA IgG (10 µg·mL^−1^), created by increasing concentrations of BSA in the inking solutions. All trends correlated well with 4-parameter logistic curves (R^2^ = 0.998). See Figure S7 for a zoomed view of both graphs in their low-concentration range.

We also explored the combination of µCP with the thiol-ene click reaction by stamping BSA proteins on glass surfaces silanized with UDTMS, and then irradiating UV light during the stamping (Figure 1C). We observed that this irradiation involves three key phenomena in the resulting structures: the magnitude of the coupling, the loss of the stamp relief, and the denaturation of the patterned proteins. As shown in Figure S6, 1 min of UV irradiation is a suitable condition for the µCP thiol-ene patterning. The desired stripped protein patterns are obtained in these conditions and display an averaged thickness (2.1 ± 0.3 nm) similar to that achieved by imine coupling (Figure 5 and Table S2). Besides, the patterns fabricated without irradiation (Figure 6, 0 min) involve negligible diffraction efficiencies, revealing minor protein physisorption on the glass surfaces treated with UDTMS.

Finally, the amount of BSA proteins patterned by physisorption, imine coupling, and thiol-ene reaction were compared. For that, increasing concentrations of BSA were employed as inking solutions, and then the diffraction efficiency of each pattern was measured (Figure 6A). At low concentrations (0–10 µg·mL^−1^), the physisorption and imine approaches displayed similar responses (Figure S7). However, for higher protein concentrations, the diffraction efficiency of the patterns fabricated by physisorption was about 2.5 and 1.5 times greater than that for imine coupling and thiol-ene reaction, respectively. These results suggest that a higher number of proteins is transferred to the substrate by µCP combined with physisorption, which also correlates with the strip thicknesses observed in Figure 5.

### 3.3. Bioanalytical Performance

Proteins can undergo significant conformational changes during the inking and stamping steps of µCP. Moreover, their native conformational structure can also be considerably altered when patterned in the host surface, even leading to functionality losses [11]. This issue can be especially critical in covalent attachment, given that the chemical composition of the proteins is also affected. This section first assesses the functionality of the BSA structures fabricated by the different µCP approximations through their binding capacity with specific antiBSA IgGs. A polyclonal whole antiserum is used in this study as antiBSA, which provides insights into the applicability of these diffractive protein patterns in biological samples.

The diffractive response after incubating specific IgGs onto patterns fabricated with different inking concentrations of protein was measured. In this experiment (Figure 6B), the diffraction efficiency is significantly higher than that observed without IgGs incubation (Figure 6A), being maximal for the patterns fabricated by passive adsorption. It may be due to the fact that albumins, such as BSA, present high immobilization strengths when physisorbed in both hydrophilic and hydrophobic surfaces [40]. These results demonstrate that these proteins keep their functionality after the patterning and they bind their target IgGs, which increases the biolayer thickness (Figure S8) and enhances the diffraction efficiency.

**Figure 7:**
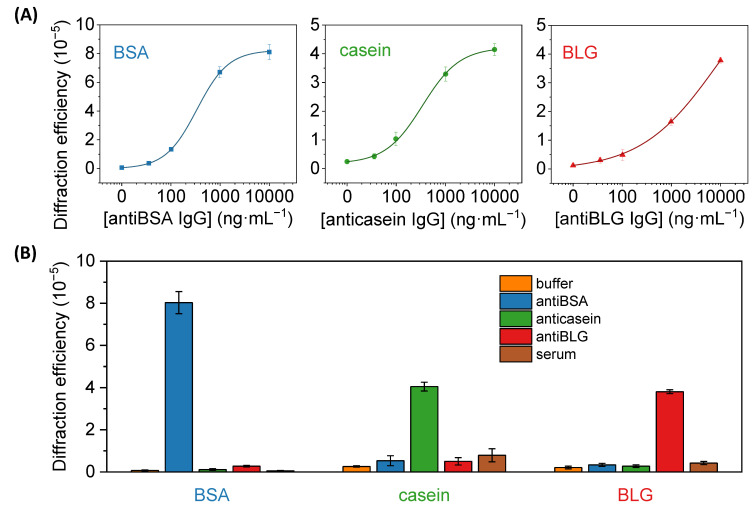
(**A**) Dose-response immunoassay curves obtained with diffractive patterns of BSA, casein, and BLG, after the incubation of a range of concentrations of specific IgG solutions (antiBSA, anticasein, and antiBLG, respectively). Experimental data were fitted to a sigmoidal regression (4-parameter logistic, R^2^ = 0.999 in all cases). See Figure S9 for a zoomed view of the graphs in their low-concentration range. (**B**) Diffraction efficiencies achieved in BSA, casein, and BLG patterns after incubating PBST (buffer), 10 µg·mL^−1^ of specific antiBSA, anticasein and antiBLG antibodies in buffer, and human serum.

These results also highlight the potential of these patterns to become diffractive transducers to quantify biorecognition events in label-free format. To explore the biosensing capabilities of this approach, the diffractive response of BSA patterns fabricated by µCP combined with passive adsorption was analyzed after incubating with increasing concentrations of specific IgGs. As shown in Figure 7, well-correlated trends were obtained in the dose-response curve of this immunoassay. A limit of detection of 30 ng·mL^−1^ and a limit of quantification of 68 ng·mL^−1^ of unlabelled IgGs, and a linear range between 68 and 870 ng·mL^−1^, are inferred from these results (Table 1).

Then, the same patterning procedure as before was applied to create diffractive gratings of the casein and BLG (Figure 2B). Those proteins are present at high concentrations in cow milk, about 32 mg·mL^−1^ for casein and 2 mg·mL^−1^ for BLG [42], being important allergens in dairy products [28,29]. Well-correlated dose-response curves in the application of these protein patterns for immunosensing specific anticasein and antiBLG IgGs are also obtained in these cases (Figure 7A). On the other hand, slightly higher limits of detection and quantification, 35 and 111 ng·mL^−1^ for anticasein together with 44 and 302 ng·mL^−1^ for antiBLG, are displayed by these systems (Table 1). This higher sensitivity obtained in the antiBSA immunoassay may be favored by the intrinsic great immobilization properties of albumins on solid substrates [40]. As observed in Table 2, representative LOD values in the state-of-the-art for the quantification of specific IgGs cover concentrations from 0.1 to 280 ng·mL^−1^ in immunoassays based on different labelling and signal development strategies. On the other hand, label-free approaches introduce important analytical advantages, while higher LODs are typically reached with these systems. The detection and quantification limits reported in this work are in the range of other recent optical immunosensing development for IgGs (Table 2), especially those that enable label-free detection. Those are promising sensitivities considering that the detection system in this study is still in its first steps of development, even though there are highly sensitive label-based and label-free approaches for the detection of specific IgGs in the state-of-the-art.

An important issue when analyzing biological samples in label-free conditions is the presence of eventual unspecific interactions (adsorption, cross-reactivity, etc) in the sensing surface. These interactions are prone to introduce undesired signal contributions that cannot be discriminated from the specific biorecognition events. To explore this phenomenon in our biosensing approach, we first evaluated potential cross-reactivities by assessing the diffractive response of the patterns upon the incubation of high concentrations (10 µg·mL^−1^) of antiBSA, anticasein, and antiBLG IgGs. As shown in Figure 7B, the incubation of anticasein and antiBLGA IgGs in BSA patterns displayed a negligible enhancement of the diffracted signals, reaching values in the same order as the one obtained after the incubation of buffer solution. Besides, the incubation of antiBSA IgG produced a substantial increment in the diffraction efficiency. In the same line, the diffraction efficiency of casein and BLG patterns was only enhanced after incubating their specific antibodies, which points out the analytical selectivity of this system.

The signal contribution due to non-specific adsorptions of undesired species that can be present in real samples was also assessed for this biosensing system. A unique feature of diffractive biosensing is the ability to minimize the signal contribution of non-specific bindings since the adsorption of non-specific species is a random process prone to take place evenly in the strips and the gaps of the protein patterns [23,43]. Therefore, even if non-specific adsorption takes place onto the protein structures, it does not increase the periodic modulation that conforms the gratings and the contribution of the non-specific binding to the diffraction efficiency is minimal. To explore this issue, we incubated pure human serum with a high concentration of non-specific species onto the protein patterns. As observed in Figure 7B, these serum incubations generated diffracted signals 0.8, 3 and 2 times higher than their corresponding incubations of blank solutions in the anti-BSA, anti-casein and anti-BLG assays, respectively. Those results offer promising insights into solving non-specific binding issues in the prospective application of these antigen patterns to analyze target biomolecules present in serum samples.

**Table 2:** Comparative table of limits of detection reported by recent optical bioanalytical developments in the scientific literature for sensing specific IgGs.

Technique	Label	Target	LOD (ng·mL^−1^)	Ref.
microarray	HRP/TMB	anticasein IgG	129	[44]
ELISA	HRP/TMB	human antiN antigen IgG	16	[45]
ELISA	HRP/TMB	human antiS antigen IgG	12.5	[45]
PMNIA^a^	Gold NPs ^a^	human antiS antigen IgG	7	[46]
microarray	HRP/TMB	human antiN antigen IgG	17	[47]
		human antiInfluenza A IgG	30	
		human antiInfluenza B IgG	280	
		human anti adenovirus IgG	110	
		Human antiRSV ^a^ IgG	12	
ELISA	HRP/TMB	anti Sap2 ^a^ IgG	0.0011	[48]
SERS-based LFIA ^a^	GERTs ^a^	human antiSARS-CoV-2 IgG	0.1	[49]
LFIA	Gold NPs ^a^	human antiEbola Virus IgG	200	[50]
LSPR ^a^	free	human antiS antigen IgG	0.08	[51]
1D photonic crystal	free	antihuman IgG	28	[52]
diffractive biosensing	free	antiBSA IgG	30	this work
diffractive biosensing	free	anticasein IgG	35	this work
diffractive biosensing	free	antiBLG IgG	44	this work

^a^ PMNIA: porous MNs and immunochromatographic assay, NPs: nanoparticles, RSV: respiratory syncytial virus, Sap2: secreted aspartyl proteinase 2, SERS: surface-enhanced Raman spectroscopy, LFIA: lateral flow immunoassay, GERTs: gap-enhanced Raman tags, LSPR: localized surface plasmon resonance.

From a general perspective, these biomolecular gratings can sense different kinds of immunoglobulins (G, E, M, and A) present in a sample. In this first approximation, the analytical results can quantify the concentration of a mix of immunoglobulin classes. However, note that this biosensing approach is compatible with the discrimination of IgGs, for example by including an additional incubation of secondary antibodies (antiIgG, antiIgE, antiIgA, etc.) in the assay. This work also introduces the basis to exploit the high versatility of µCP to fabricate patterns of a broad range of biomolecules. For example, diffractive structures of antibodies can be patterned by µCP to detect the presence of allergens in dairy products. This configuration should take into account the potential activity loss undergone by antibodies when patterned by µCP, together with the introduction of alternative stamping strategies to overcome this issue [11].

## 4. Conclusions

This investigation focuses on submicron patterns of allergen proteins created by different microcontact printing (µCP) chemistries and their application to detect antibodies involved in dairy allergies. The study demonstrates that exposing the PDMS stamps to UV-ozone before the inking may compromise their performance when patterning at the submicron scale. Moreover, the conditions required to pattern organosilanes by µCP also damage the relief of the stamp, whereas marrying µCP with physisorption, imines reaction, and thiol-ene coupling is a successful strategy to pattern proteins at this scale. In the thiol-ene approach, the irradiation time is a critical parameter to reach maximal couplings, keep the pattern structure, and avoid protein denaturation. Homogeneous patterns of periodic protein strips (about 280 nm wide and 2–3 nm tall) are obtained in all the cases, which present great potential as diffractive transducers for label-free biosensing. Functional submicron patterns of allergen proteins involved in cow milk allergy can be created and used to sense specific immunoglobulins G in solution. In particular, this work provides insights into their implementation in bovine serum albumin, casein, and β-lactoglobulin, displaying limits of detection of 30, 35, and 44 ng·mL^−1^, respectively. In addition to IgGs, these results introduce the basis for the prospective fabrication and application of these diffractive structures to sense other immunoglobulins and macromolecules involved in dairy and other food allergies.

## Figures and Tables

**Table 1:** Limits of detection and quantification calculated from the experimental trends.

IgG	LOD (ng·mL^−1^)	LOQ (ng·mL^−1^)	Linear Range (ng·mL^−1^) *
antiBSA	30	68	68–425
anticasein	35	111	111–450
antiBLG	44	302	302–1525

* see Table S3 for the linear relationships between the antigens and the antibodies.

## Data Availability

The data presented in this study are available on request from the corresponding author. The data are not publicly available due to privacy restrictions.

## Supplementary Materials

The following supporting information can be downloaded at: https://www.mdpi.com/article/10.3390/bios13020214/s1, Figure S1. Static contact angle values of glass substrates (A) irradiated with UV-light for increasing times and (B) treated with increasing concentrations of 10-undecenyltrimethoxysilane after the ozone activation; Figure S2. Static contact angles values measured after each functionalization step for the imine coupling; Figure S3. FESEM images of the PDMS grooved structure after different UV-ozone exposure times. Scale bars correspond to 2 µm, Figure S4. Diffraction efficiency and FESEM images of the PDMS stamps after their immersion in water and toluene for 160 min, and after their exposition to chemical vapor deposition (CVD) of APTES; Figure S5. Diffraction efficiency measured after incubating different concentrations of antiBSA IgG in PBS-T onto BSA patterns unblocked (grey) and blocked (blue) with ethanolamine, fabricated through the imine route; Figure S6. Diffraction efficiency (black squares and continuous line) and fluorescence signals (blue dots and dashed line) of BSA patterns after incubating fluorophore-labeled antiBSA (10 µg·mL^−1^), fabricated by µCP coupled to thiol-ene reaction by increasing the UV-irradiation times; Figure S7. Zoomed view of the low concentration range of both representations in Figure 6 of the main manuscript; Figure S8. AFM images of BSA patterns after incubating a solution of antiBSA in PBS-T at (A) 0 µg·mL^−1^ and (B) 10 µg·mL^−1^, and (C) the corresponding height of the protein strips measured from these scans; Figure S9. Zoomed view of the low concentration range of the graphs presented in Figure 7 of the main manuscript; Table S1. Characterization results of the PDMS stamps after different ozone exposure times; Table S2. Structural parameters measured from the AFM images in Figure 5; Table S3. Parameters of the linear fittings employed to infer the linear range of the immunoassays (Diffraction efficiency = a + b [IgG]).
