# Supplementary material for: Patterned Biolayers of Protein Antigens for Label-Free Biosensing in Cow Milk Allergy
Source: Biosensors (Basel). 2023 Feb 1;13(2):214. doi: 10.3390/bios13020214 (PMC9953870; doi:10.3390/bios13020214)
Supplement: Supplementary file 1 [file biosensors-13-00214-s001.zip › biosensors-2182242-supplementary.pdf]

biosensors

Supplementary Material

Patterned Biolayers of Protein Antigens for Label-free Biosensing in Cow Milk Allergy

Augusto Juste-Dolz, Estrella Fernández, Rosa Puchades, Miquel Avella-Oliver and Ángel Maquieira

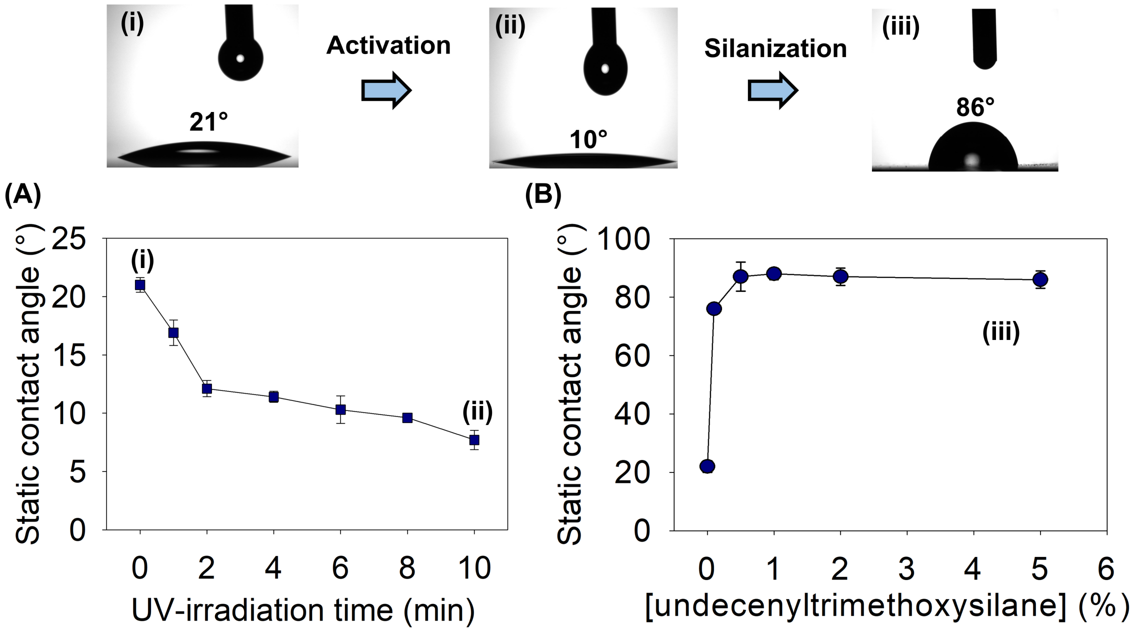

Figure S1. Static contact angle values of glass substrates **(A)** irradiated with UV-light for increasing times and **(B)** treated with increasing concentrations of 10-undecenyltrimethoxysilane after the ozone activation.

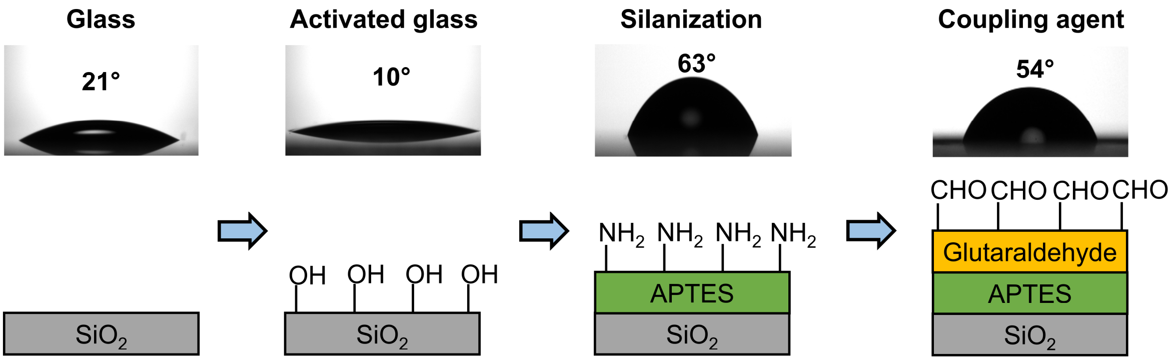

Figure S2. Static contact angles values measured after each functionalization step for the imine coupling.

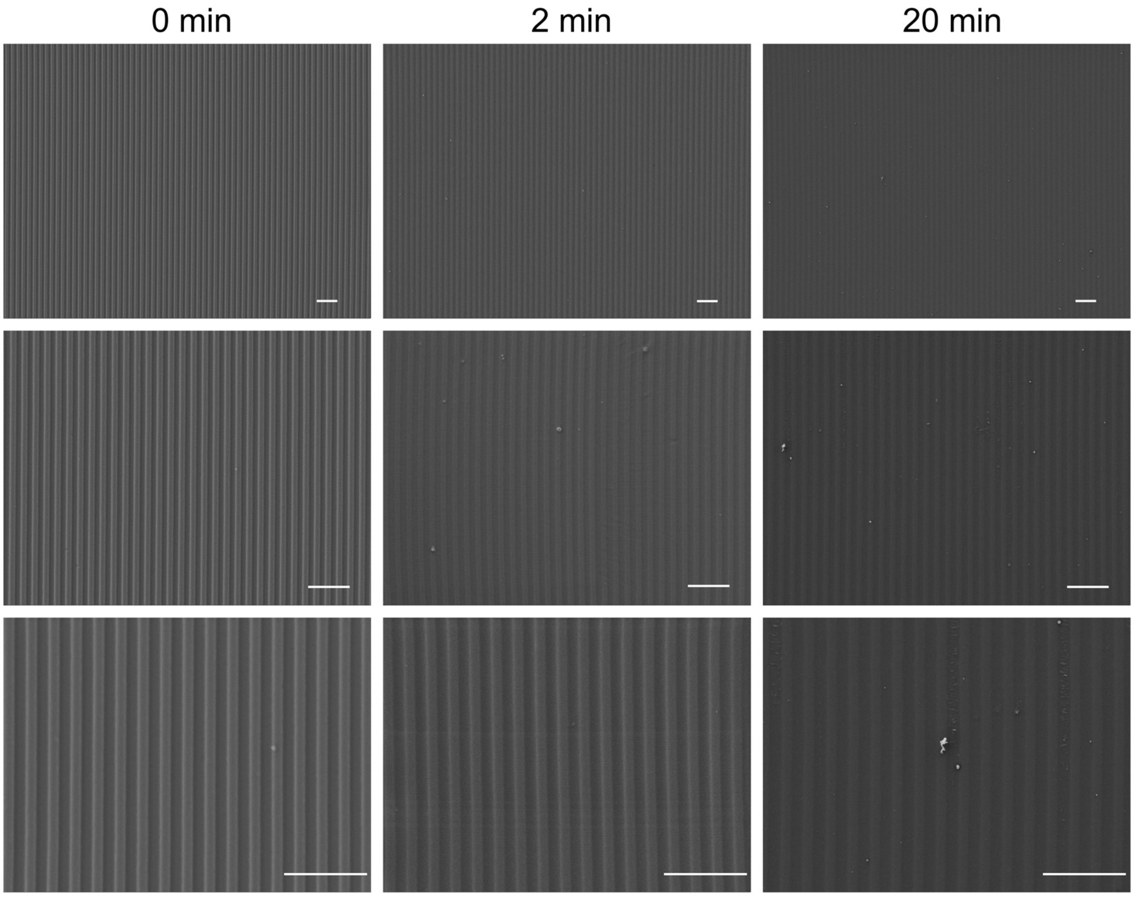

Figure S3. FESEM images of the PDMS grooved structure after different UV-ozone exposure times. Scale bars correspond to 2 μm.

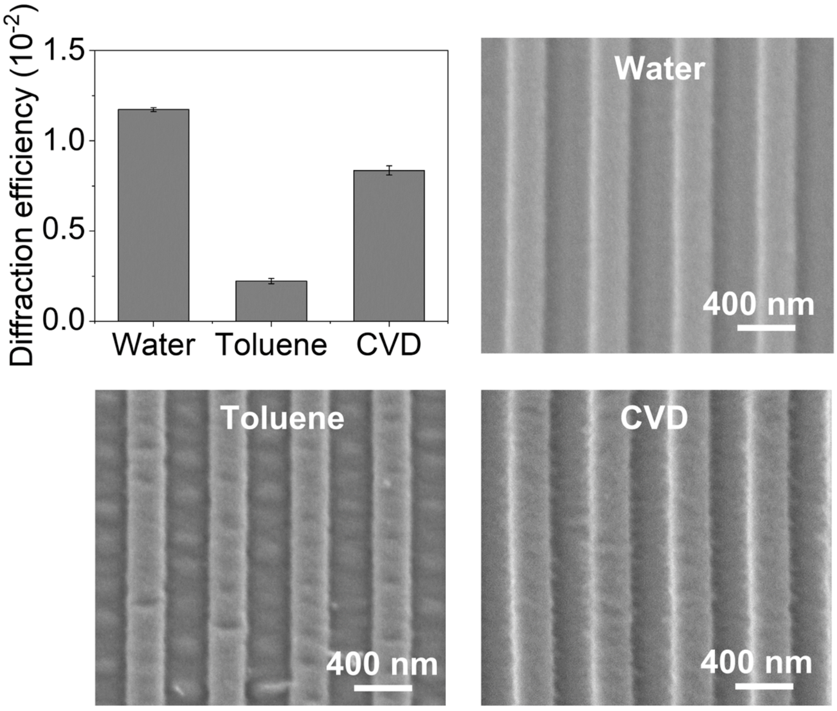

Figure S4. Diffraction efficiency and FESEM images of the PDMS stamps after their immersion in water and toluene for 160 min, and after their exposition to chemical vapor deposition (CVD) of APTES.

Organosilanes need to be diluted and solved in organic and aprotic solvents (typically toluene) that avoid their polymerization (*Anal. Bioanal. Chem. 2021, 414, 5071-5085*). But the results in Figure S4 show that this incubation introduces structural heterogeneities that lead to a significant drop in the diffraction efficiency (from 1.22 to 0.22) compared to the incubation of water used as a control.

Chemical vapor deposition (CVD) is a solventless alternative also commonly used to create monolayers of organolsilanes, where the surface to be silanized is directly exposed to the vapor of the selected organosilane (*Langmuir 2018, 34, 1400-1409*). However, the resulting stamps also display structural heterogeneities in the FESEM images as well as an important decrease in the diffraction efficiency.

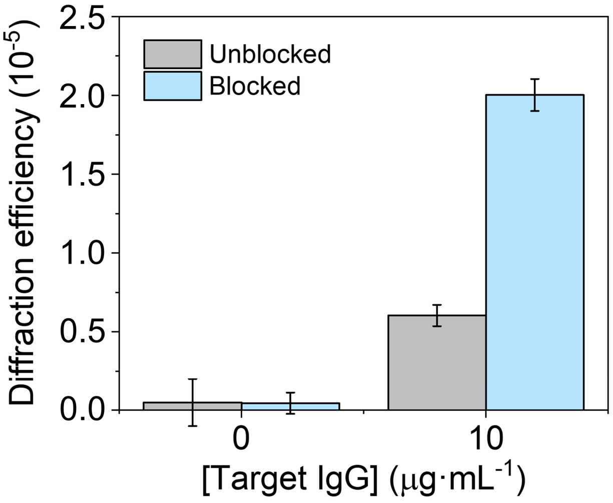

Figure S5. Diffraction efficiency measured after incubating different concentrations of antiBSA IgG in PBS-T onto BSA patterns unblocked (grey) and blocked (blue) with ethanolamine, fabricated through the imine route.

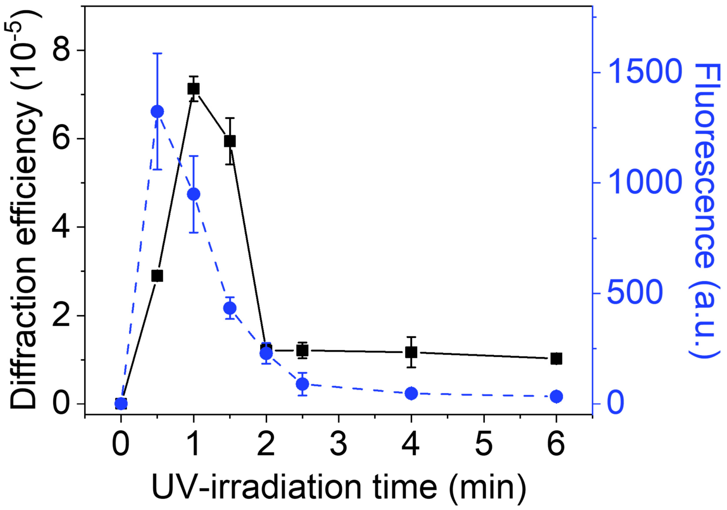

Figure S6. Diffraction efficiency (black squares and continuous line) and fluorescence signals (blue dots and dashed line) of BSA patterns after incubating fluorophore-labeled antiBSA (10 μg·mL^{-1}), fabricated by μCP coupled to thiol-ene reaction by increasing the UV-irradiation times.

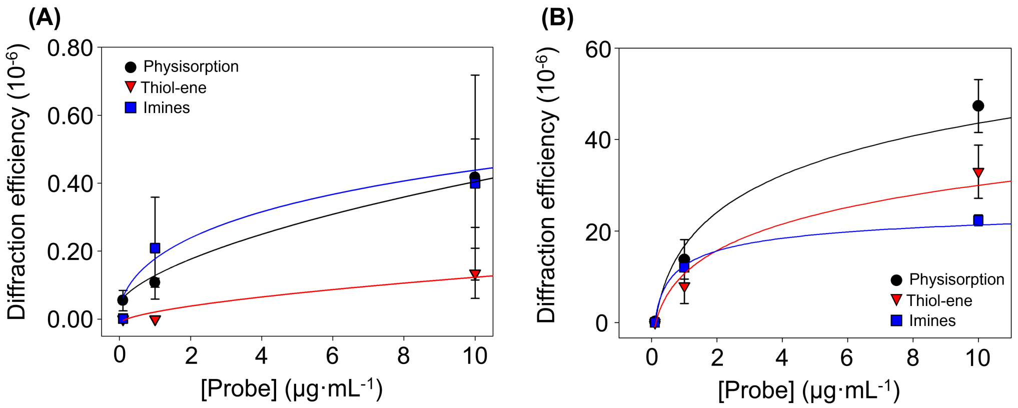

Figure S7. Zoomed view of the low concentration range of both representations in Figure 6 of the main manuscript.

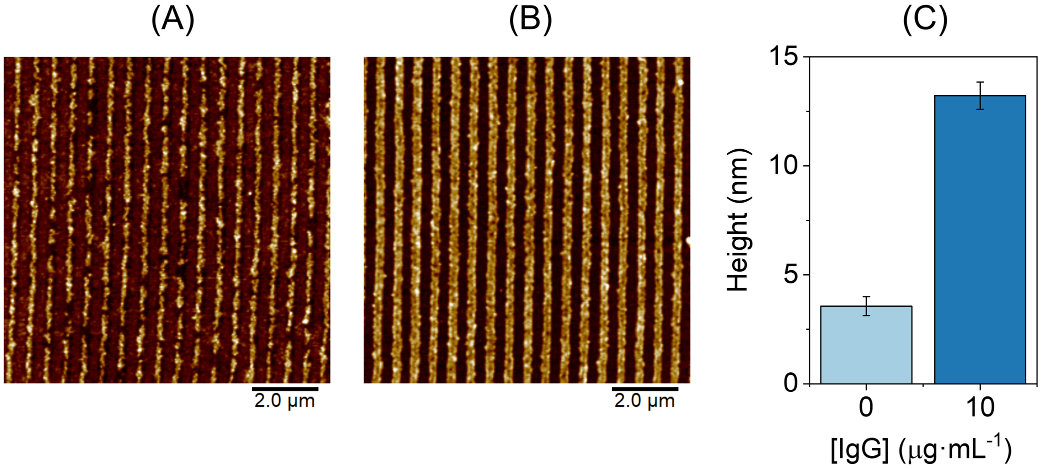

Figure S8. AFM images of BSA patterns after incubating a solution of antiBSA in PBS-T at (A) 0 μg·mL-1 and (B) 10 μg·mL-1, and (C) the corresponding height of the protein strips measured from these scans.

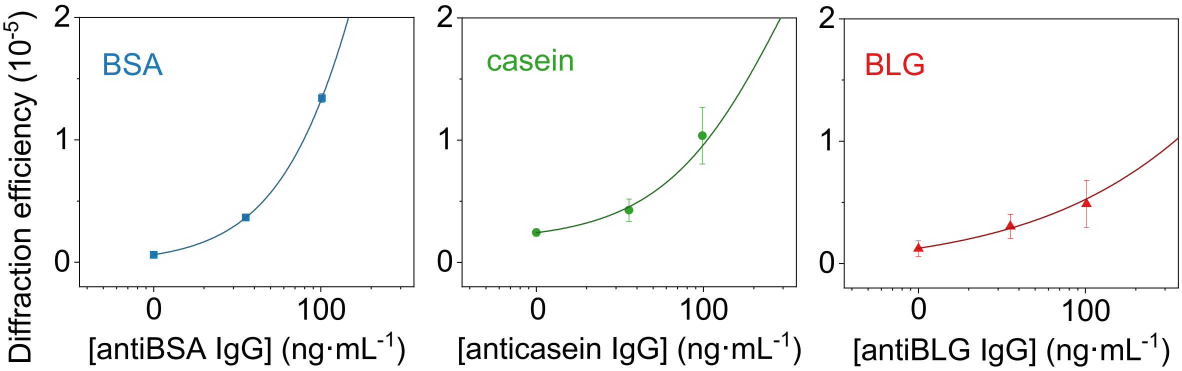

Figure S9. Zoomed view of the low concentration range of the graphs presented in Figure 7 of the main manuscript.

Table S1. Characterization results of the PDMS stamps after different ozone exposure times.

Ozone exposure time (min)	0	2	20
Diffraction efficiency ($\cdot 10^{-3}$)	7.5 ± 0.4	4.8 ± 0.1	2.3 ± 0.3

**Static contact angle (°)**	130 ± 2	122 ± 1	87 ± 3
**Pattern period (nm) ***	563 ± 4	557 ± 2	544 ± 3
**Duty cycle ***	50 ± 1	53 ± 1	54 ± 1
**Groove height (nm) ***	99 ± 1	72 ± 6	39 ± 6

* Measured from AFM scans (Figure 3C).

Table S2. Structural parameters measured from the AFM images in Figure 5.

Strategy	Period (nm)	Duty cycle (%)	Height (nm)
**Physisorption**	564 ± 8	43 ± 2	3.3 ± 0.4
**Imines**	580 ± 10	58 ± 3	2.0 ± 0.3
**Thiol-ene**	582 ± 7	60 ± 3	2.1 ± 0.2

Table S3. Parameters of the linear fittings employed to infer the linear range of the immunoassays (*Diffraction efficiency = a + b [IgG]*).

	**a**	**b**	**R^2**
**BSA**	$3 \cdot 10^{-6}$	$1 \cdot 10^{-7}$	0.99
**casein**	$6 \cdot 10^{-6}$	$4 \cdot 10^{-8}$	0.99
**BLG**	$7 \cdot 10^{-6}$	$9 \cdot 10^{-9}$	0.99
